# Biosafety risk assessment of gold and aluminum nanoparticles in tumor-bearing mice

**DOI:** 10.1063/5.0144481

**Published:** 2023-03-23

**Authors:** Ge Zhu, Zhihan Li, Yuning Zhang, Xiandi Meng, Meng Guan, Zheng Hu, Yong-Guang Yang, Kun Liu, Tianmeng Sun

**Affiliations:** 1 Key Laboratory of Organ Regeneration and Transplantation of Ministry of Education, Institute of Immunology, The First Hospital, Jilin University, Changchun, Jilin, China; 2 National-Local Joint Engineering Laboratory of Animal Models for Human Diseases, Changchun, Jilin, China; 3 State Key Laboratory of Supramolecular Structure and Materials, College of Chemistry, Jilin University, Changchun, Jilin, China; 4 International Center of Future Science, Jilin University, Changchun, Jilin, China

## Abstract

To improve the biosafety of the nanodelivery system, this study developed novel monodisperse spherical aluminum nanoparticles (Al NPs) and evaluated their cytotoxicity *in vitro* and distribution and biotoxicity *in vivo*. Compared with gold nanoparticles of the same size, Al NPs not only had low cytotoxicity *in vitro* but also did not cause accumulation in major organs *in vivo* after intravenous injections. No significant abnormalities were observed in the serum biochemical indices of mice injected with Al NPs. Additionally, no substantial changes occurred in the histopathology of major organs, and no apparent biological toxicity was measured after consecutive injections of Al NPs. These results indicate that Al NPs have a good biological safety and provide a new method for developing low-toxicity nanomedicine.

## I. INTRODUCTION

Nanotechnology has developed rapidly and is currently utilized in many areas of biomedical science, such as drug delivery, bioimaging, and tissue engineering.^1–3^ Although nanomedicine is generally considered a promising field of diagnosis and treatment, the biodistribution and toxicology of novel nanoparticles must be carefully assessed before clinical use. Metal-based nanomaterials have unique physical and chemical properties and play an essential role in drug delivery systems susceptible to cellular efflux pumps.^4–6^ The optical, electronic, and catalytic properties of different metallic elements affect cellular uptake, biological distribution, retention, clearance pathways, and toxicity. Understanding the characteristics of different metallic materials and their biological distribution can help to optimize nanoparticle function. Knowledge of these characteristics is essential for treating tumors. However, as the types and applications of metal-based nanomaterials increase, relevant information on potential toxicity hazards is desperately required.

The tumor accumulation of nanoparticles is mainly based on the enhanced permeability and retention (EPR) effect because of the different pathophysiological characteristics between tumors and healthy tissues. Defective vasculature with high permeability exists in most solid tumors, allowing for the extravasation of materials with sizes up to hundreds of nanometers.^7^ The absence of lymphatic drainage leads to a relatively effective and selective accumulation of nanoparticles in tumors.^8,9^

Gold nanoparticles (Au NPs) have recently been applied in many fields, such as pharmaceutical drug carriers, bioimaging, and photothermal therapy.^10–12^ However, as soon as Au NPs were first used in medicine, questions were raised about the pharmacokinetics, biodistribution, and toxicity to the organism.^13^ Previous studies showed that these particles could accumulate and persist in the organism for decades, causing unexpected radiation effects.^14,15^ The cytotoxicity depended on the size, concentration, surface properties of Au NPs, and the duration effect on cells. Additionally, the primary mechanism of cytotoxicity caused by Au NPs involved the damage of vacuoles, the release of these Au NPs into the cytoplasm disrupted normal cell function, inhibition of cell proliferation, intracellular calcium release, nitrogen oxide, and oxidative stress.^13,16^ *In vivo*, upregulation of genes via intravenous injection of Au NPs is involved in apoptosis, response to stress, cell cycle, inflammation, signal transduction, and metabolism.^17^ Au NP-induced fatigue, loss of appetite, change of fur color, weight loss, and even death have been observed in mice. Pathological examination indicates an increased number of Kupffer cells in the liver, diffusion of white pulp in the spleen, and loss of structural integrity in the lung.^18^

Aluminum is the most predominant metal in the Earth's crust (∼8.8%),^19^ which means it may be more commercially viable compared with Au NPs. The applications of aluminum nanoparticles (Al NPs) in medicine have been tested worldwide. In an organism, aluminum is mainly eliminated through urinary excretion, and over half of the injected aluminum was left in the bloodstream after 8 h.^19,20^ Encephalopathy has never been reported, although neurotoxicity may be observed after the administration of aluminum salts. There is consensus in clinical toxicology that this metal toxicity is associated with increased levels in biological media. The potential neurotoxicity has only been observed in cases of renal failure and excessive accumulation of aluminum in the brain.^21^ However, aluminum levels in vaccinated people's blood are similar to those without occupational exposure to aluminum (15 *μ*g/l), and neurotoxicity related to treatment containing aluminum salts has never been reported in patients with normal renal function.^22^ The nontoxic nature of aluminum and the EPR effect provides additional benefits to enable easy penetration and accumulation of drugs at the tumor sites.

The tumor-bearing condition can trigger physiological and pathological changes, promote vascular permeability, and remodel the immune system.^23,24^ Our work further revealed remarkable plasticity in the systemic immune state.^25,26^ The special tumor-bearing state can modify the distribution profile of systemically injected nanoparticles.^27^ Conversely, the injection of nanoparticles might adjust the immune macroenvironment.^28^ To develop novel low-toxicity nanomedicine, the distribution and biotoxicity of nanoparticles need to be well studied. This information can guide the appropriate particle selection under different disease conditions. Thus, we aimed to develop novel monodisperse spherical Al NPs and comprehensively study the biodistribution and biotoxicity under tumor-bearing conditions, as well as provide strategies for tumor drug delivery system.

## II. RESULTS AND DISCUSSION

### A. Preparation and characterization of Au NPs and Al NPs

**FIG. 1.**
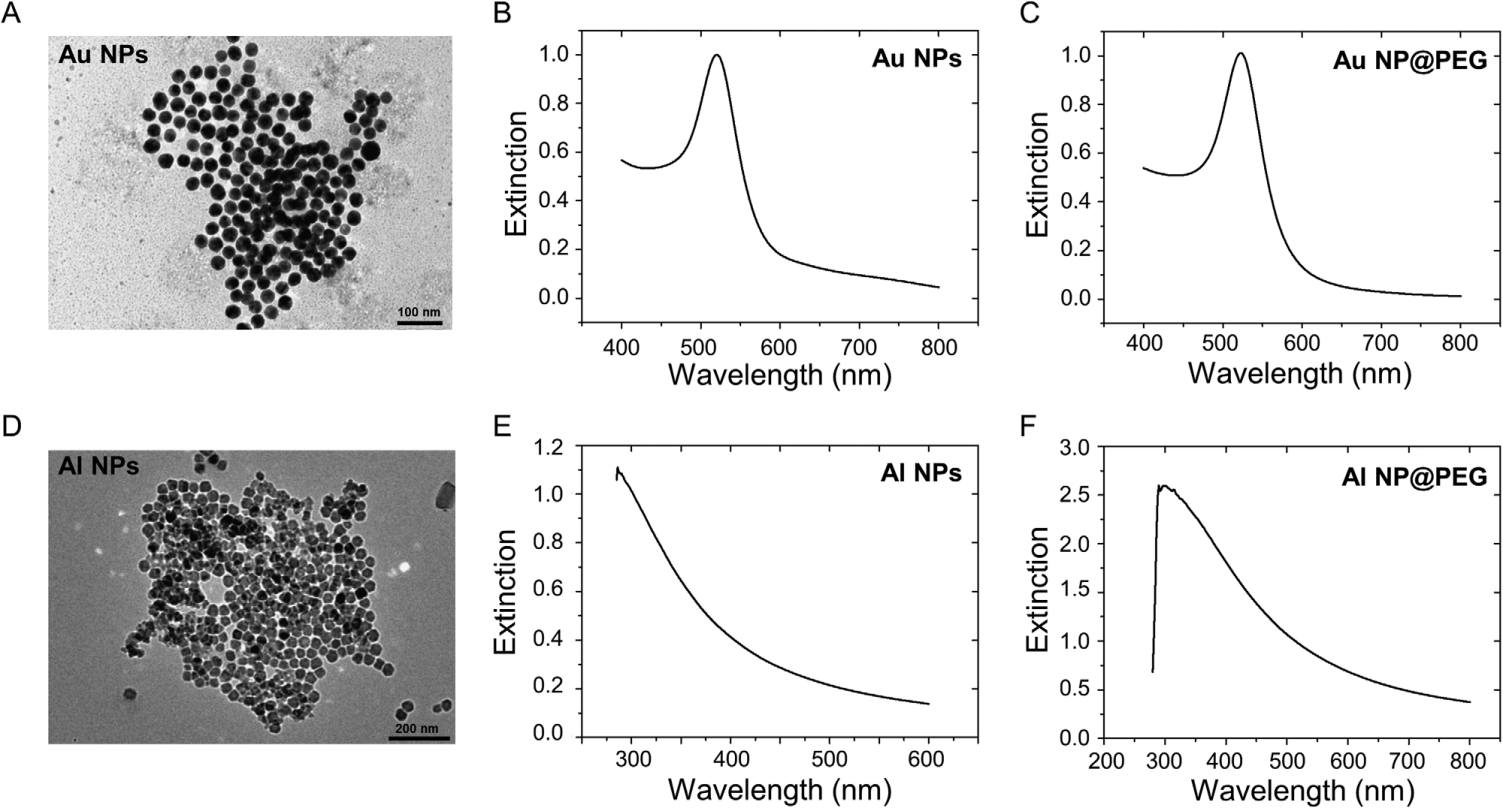
Characterization of Au NPs and Al NPs. Representative TEM image (a) and extinction spectrum (b) of Au NPs. (c) Extinction spectrum of Au NP@PEG. Representative TEM image (d) and extinction spectrum (e) of Al NPs. (f) Extinction spectrum of Al NP@PEG.

We successfully synthesized Au NPs and Al NPs in this study. The hydrodynamic diameters were 30.6 ± 2.2 nm for Au NPs and 33.2 ± 4.3 nm for Al NPs (Fig. S1). The zeta potentials for Au and Al NPs were −20.6 ± 0.6 and 11.5 ± 0.6 mV, respectively. Transmission electron microscope (TEM) analysis revealed that the monodisperse and uniform Au and Al NPs were spherical geometry structures [Figs. 1(a) and 1(d)]. The extinction spectra of the Au and Al NPs showed that their full width at half-maximum of the extinction band were narrow, indicating that the nanoparticles were monodisperse [Figs. 1(b) and 1(e)]. The slight redshift of the extinction spectrum of polyethylene glycol (PEG) grafted nanoparticles indicated that the PEG was grafted on the nanoparticle surfaces [Figs. 1(c) and 1(f)].

### B. Cellular uptake and cytotoxicity of Au NPs and Al NPs

**FIG. 2.**
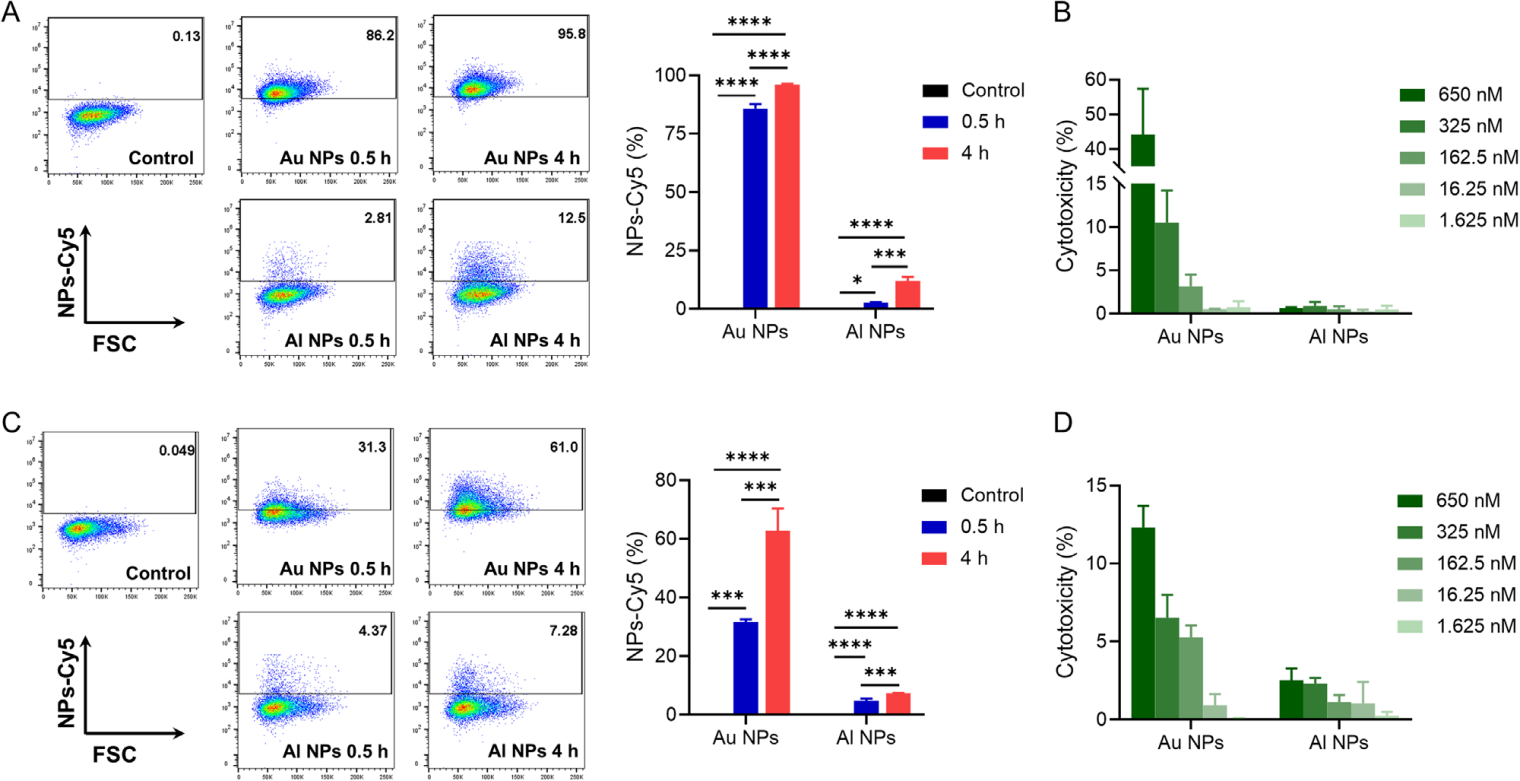
Cellular uptake and cytotoxicity of nanoparticles. (a) Representative flow cytometry plots and frequencies of Cy5-nanoparticle uptake by B16 cells. (c) Representative flow cytometry plots and frequencies of Cy5-nanoparticle uptake by NCTC1469 cells. Cytotoxicity of Au and Al NPs on B16 cells (b) and NCTC1469 cells (d). Data are presented as mean ± SD (n = 3 per group). ^*^P < 0.05, ^***^P < 0.001, and ^****^P < 0.0001.

To compare cellular uptake of Au and Al NPs, we determined the delivery efficacy in a mouse melanoma cell line (B16) and liver normal parenchyma cell line (NCTC1469) *in vitro* using fluorescent dye Cy5-labeled NPs (Cy5-Au NPs and Cy5-Al NPs). We incubated B16 or NCTC1469 cells with Cy5-labeled NPs for 30 min and 4 h. Flow cytometry revealed that Cy5-positive cells increased significantly in B16 and NCTC1469 cells after co-incubation with Cy5-Au NPs. In particular, B16 cells were incubated with Cy5-Au NPs for 4 h, and the proportion of Cy5-positive cells was close to 100%. However, when the previous two types of cells were co-incubated with Cy5-Al NPs, the proportion of Cy5-positive cells decreased significantly compared with the Cy5-Au NP group cells. When the co-incubation time was extended to 4 h, the proportion of Cy5-positive cells in B16 and NCTC1469 cells incubated with Cy5-Al NPs was less than 15% and 10%, respectively [Figs. 2(a) and 2(c)].

Based on the cellular uptake assay, to determine whether Au and Al NPs had different cytotoxicity *in vitro*, we performed a lactate dehydrogenase (LDH) assay. Figures 2(b) and 2(d) show that the cellular killing rate of Au NPs on B16 cells was close to 50% at 650 nM, and the cytotoxicity decreased with the gradually decreasing nanoparticle concentration. That is, the cytotoxicity was positively correlated with the Au NP concentration. The same trend was observed in the NCTC1469 cells toxicity assay. However, after incubation with Al NPs for 24 h, mouse melanoma and normal liver cells showed good cellular activity. These results were consistent with the trend of cellular uptake experiments, indicating that Al NPs had insignificant intracellular accumulation and little cytotoxicity.

### C. Biodistribution and accurate quantification of nanoparticle retention within tissues

**FIG. 3.**
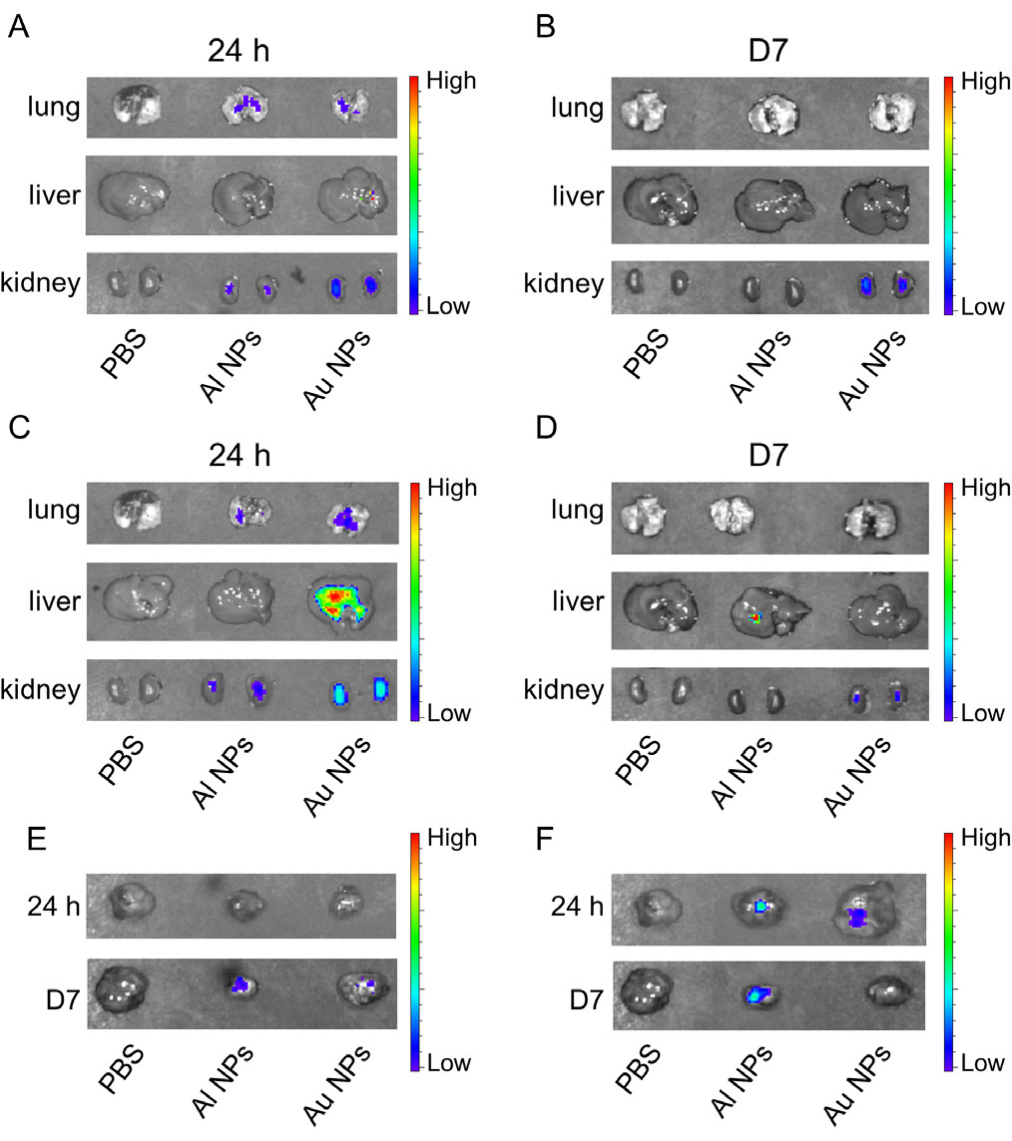
Biodistribution of Au NPs and Al NPs *in vivo*. Fluorescence images of the lung, liver, and kidney at 24 h (a) and 7 days (b) after a single injection of Cy5-NPs. Fluorescence images of the lung, liver, and kidney at 24 h (c) and 7 days (d) after three consecutive injections of Cy5-NPs. Fluorescence images of the tumor after a single (e) or three consecutive injections (f) of Cy5-NPs.

To determine the biodistribution of Au and Al NPs, we used the Cy5-labeled NPs with a murine melanoma tumor model created by injecting B16 cells subcutaneously in C57BL/6 mice. The Cy5 fluorescent signal was detected in the liver and kidneys 24 h after a single injection of Au NPs, compared with that of control or Al NPs [Fig. 3(a)]. At 24 h after three consecutive injections of Au NPs, the Cy5 fluorescent signal in liver and kidneys was stronger than that of a single injection. However, the weak fluorescent signal could be detected only in the kidneys of the Al NP group mice [Fig. 3(c)]. At 7 days after the NP injection, the Cy5 fluorescent signal was detected only in the kidneys of the Au NP group [Figs. 3(b) and 3(d)]. At 7 days after a single injection of Cy5-NPs, the Cy5 fluorescence signal was observed in the tumor of the Al NP group [Fig. 3(e)]. Under the condition of three consecutive injections, the Cy5 fluorescence signal was detected in the tumor of the Al NP group at 24 h after injections, which was stronger than that of the Au NP group. The Cy5 fluorescence signal was still detected in tumor of the Al NP group at 7 days after injections [Fig. 3(f)]. These results indicated that intravenous injection of Au NPs would cause nanoparticle accumulation in the lung, liver, and kidneys *in vivo*, but this phenomenon is not observed for Al NPs. Nevertheless, the intravenous injected Al NPs effectively accumulated in tumors.

**FIG. 4.**
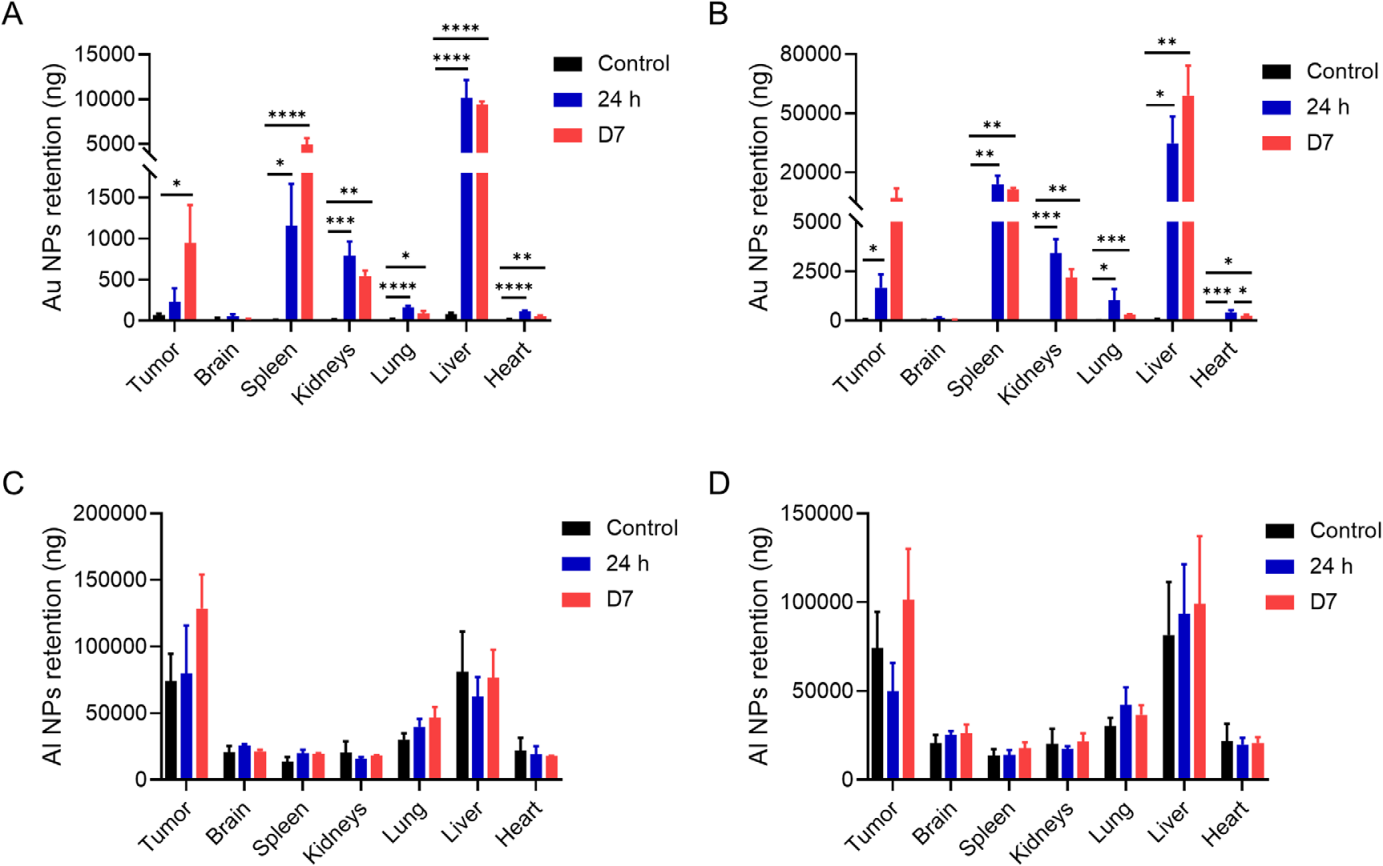
ICP-MS analysis demonstrates the organ and tumor distribution of injected nanoparticles in tumor-bearing mice. The retention of Au NPs in major organs and tumors after a single (a) or three consecutive injections (b). The retention of Al NPs in major organs and tumors after a single (c) or three consecutive injections (d). Data are presented as mean ± SD (n = 3 per group). ^*^P < 0.05, ^**^P < 0.01, ^***^P < 0.001, and ^****^P < 0.0001.

For the biodistribution and toxicology assay, one of the main tasks is accurately determining the content and localization of materials in the examined tissues. Instrumental neutron activation analysis (INAA) and inductively coupled plasma–mass spectrometry (ICP-MS) are the “gold standards” for quantifying tissue particle concentrations.^29,30^ To compare the retention of Au and Al NPs *in vivo*, the tumors and major organs (brain, spleen, kidneys, lung, liver, and heart) of B16 tumor-bearing mice intravenously injected with nanoparticles were quantitatively detected using ICP-MS. At 24 h after a single injection of Au NPs, the accumulation of Au NPs in tumor-bearing mice was detected in major organs except for the brain, including the spleen, kidneys, lung, liver, and heart, compared with the control group [Fig. 4(a)]. Specifically, the retention of Au NPs in the spleen and liver was significantly increased. In healthy mice, most administered nanoparticles were retained by the lungs, liver, and spleen, as previously published.^31^ The content of Au NPs in tumors was also increased, which may be related to the EPR effect mentioned above.^7,24^ Quantitative detection of Au NPs was performed again in major organs and tumors on the seventh day after injection, and the retention of Au NPs did not decrease significantly compared with that of 24 h after injection. However, it increased in the spleen, resulting from further uptake of nanoparticles by the mononuclear phagocytic cell system in spleen.^32^ After three consecutive injections, the accumulation of Au NPs in major organs further increased compared with that after a single injection [Fig. 4(b)]. This indicated that it was challenging to metabolize Au NPs, and that the accumulation *in vivo* was one of the important causes of toxicity of Au NPs. The B16 tumor-bearing mice were intravenously injected with Al NPs of the same size by the same injection method. The results of ICP-MS showed that the content of Al NPs in major organs of mice was not statistically different from that in the control group. This was independent of whether it was a single injection [Fig. 4(c)] or three consecutive injections [Fig. 4(d)]. This indicated no apparent accumulation of Al NPs *in vivo*, which provided a theoretical basis for the biosafety of Al NP biological application.

### D. *In vivo* toxicity of Au NPs and Al NPs

**FIG. 5.**
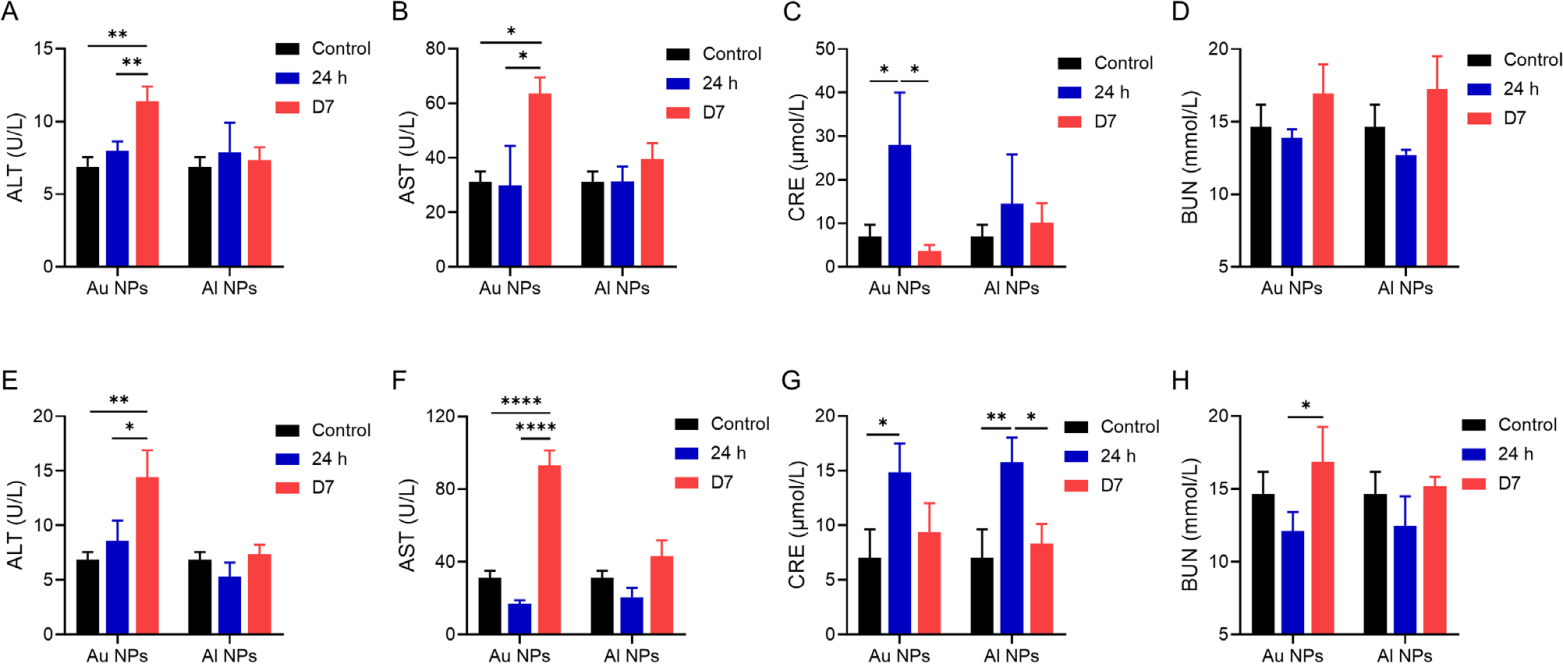
Results of serum biochemical analyses from mice injected with PBS and NPs. The results of ALT (a), AST (b), CRE (c), and BUN (d) of serum from mice after a single injection. The results of ALT (e), AST (f), CRE (g), and BUN (h) of serum from mice after three consecutive injections. Data are presented as mean ± SD (n = 3 per group). ^*^P < 0.05, ^**^P < 0.01, and ^****^P < 0.0001.

Serum biochemical indices, including liver and kidney function, are essential for revealing nanoparticle biotoxicity. To compare the differences in biosafety between Au and Al NPs, we analyzed alanine aminotransferase (ALT) and aspartate aminotransferase (AST) for liver function and blood urea nitrogen (BUN) and creatinine (CRE) of serum for kidney function. Serum ALT and AST values at 24 h after a single intravenous injection of Au NPs showed no significant changes compared with the control group, but they increased on the seventh day after injection. There were no apparent abnormalities in serum ALT and AST at 24 h and 7 days after a single intravenous administration of Al NPs [Figs. 5(a) and 5(b)]. Figures 5(e) and 5(f) show that serum ALT and AST values increased further on the seventh day after three intravenous injections of Au NPs, while there was no obvious abnormality after injections of Al NPs for three consecutive days, indicating that the accumulation of Au NPs would cause damage to liver function to a certain extent. After a single or three consecutive injections of Au and Al NPs, there was a transient increase in CRE values of serum at 24 h, but it remained within the normal range [Figs. 5(c) and 5(g)], and there were no significant changes in BUN values [Figs. 5(d) and 5(h)]. These results fully demonstrate the biosafety of Al NPs.

**FIG. 6.**
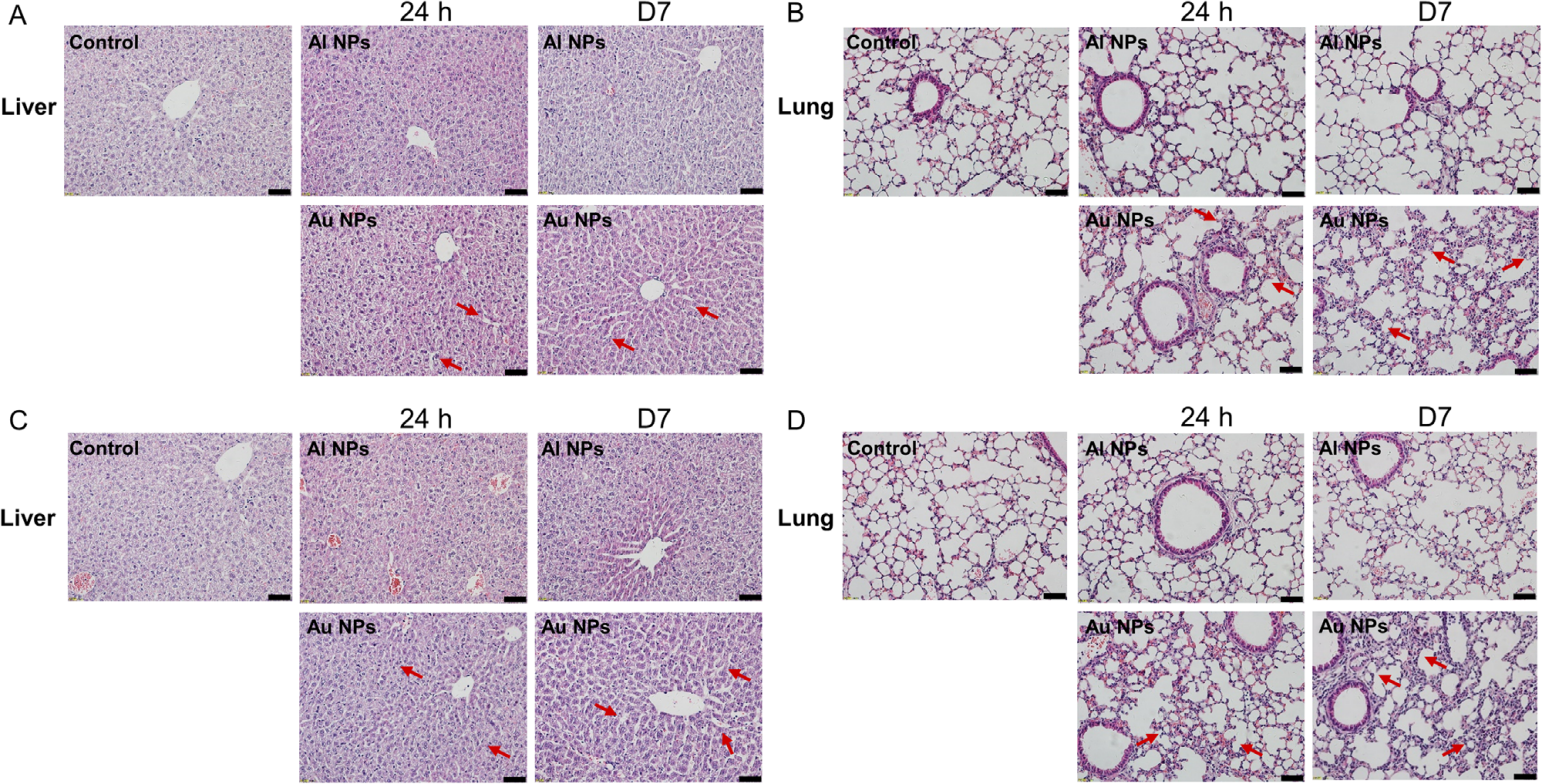
Representative micrographs of liver and lung in mice. H&E staining of the liver (a) and lung (b) from the control, Au, and Al NP groups after a single injection. H&E staining of the liver (c) and lung (d) from the control, Au, and Al NP groups after three consecutive injections (magnification 200×, scale bar = 50 *μ*m).

Hematoxylin-eosin (H&E) staining was further performed on major organs and tumors of mice. Hepatocytes are organised into plates, separated by vascular channels (sinusoids). Normally, mammalian hepatocyte plates are only the thickness of one cell. However, the plates of the Au NP group were thicker than those of the control group. Hepatocyte swelling, nuclear deep staining, and disordered arrangement were found in the liver tissue of the Au NP group, especially in the group of three consecutive injections of Au NPs [Figs. 6(a) and 6(c)], which indicated injury of the liver. In the lung, light interalveolar septal thickening and inflammatory cell infiltration was observed in all Au NP injected mice, while the Al NP group mice had normal lung tissue morphology [Figs. 6(b) and 6(d)]. Finally, no significant abnormalities were observed in other vital organs (heart, brain, spleen, and kidney) and tumors compared with the control group (Fig. S2).

## III. CONCLUSION

This study involved the monitoring (24 h and 7 days) of mice that received Au and Al NPs intravenously. An overall biosafety assessment of both Au and Al NPs in the mice was obtained by combining a cytotoxicity assay *in vitro* and serum biochemistry profiles, biodistribution, ICP-MS quantification, and histopathological analysis *in vivo*. For the *in vitro* experiments, the internalization rate of Au NPs in mouse melanoma and normal liver cells was stronger compared with those of Al NPs. Therefore, the cytotoxicity of Au NPs was also much higher compared with that of Al NPs. Concerning biological distribution, Au NPs were accumulated in the liver and spleen of the mice, causing pathological changes in the liver and lung. Simultaneously, no accumulation of Al NPs had been detected in the major organs *in vivo* after intravenous injection, nor did the NPs cause significant changes in liver and kidney function or pathological changes in major organs. Overall, the good biological safety profile of Al NPs was verified, and this study will provide new insights into nanoparticle biodistribution and biotoxicity. Moreover, this work allows for the improved development of nanomedicines incorporating Al NPs for treating tumors.

## IV. METHODS

### A. Materials

Cy5 N-hydroxy succinimide ester (Cy5-HNS) was obtained from Aladdin Biochemical Technology Co., Ltd. (Shanghai, China). Chloroauric acid monohydrate (HAuCl_4_), sodium citrate, and ascorbic acid were purchased from Sigma-Aldrich (St. Louis, Missouri, USA). Thiol-terminated poly(ethylene oxide) (*M*_n_ = 5000 g/mol) and carboxyl-terminated poly(ethylene oxide) (*M*_n_ = 5000 g/mol) were obtained from ToYong Biotechnology Co., Ltd. (Shanghai, China). The mouse melanoma cell line (B16) and liver normal parenchyma cell line (NCTC1469) were purchased from American Type Culture Collection (ATCC).

### B. Preparation of Al NPs and Au NPs

Spherical Au NPs (30.6 nm) were synthesized using the regrowth method reported previously.^33^ The seed solution was prepared using the citrate reduction method. Specifically, the HAuCl_4_ (1.2 ml, 20 mM) and sodium citrate (2.0 ml, 2.0% w/v) were added into 96.8 ml H_2_O and boiled for 15 min. Then the precursor solution A, containing AuCl_4_ (5.0 ml, 0.40% w/v), and the reducing solution B, containing sodium citrate (2.5 ml, 0.50% w/v) and ascorbic acid (2.5 ml, 1.0% w/v), were added separately via a peristaltic pump under stirring. Finally, the solution was kept boiling for 30 min and cooled down.

We synthesized monodisperse spherical Al NPs (33.2 nm) by catalytic reduction of precursor H_3_Al, using PEG as polydentate surface ligands. H_3_Al was synthesized according to previously reported protocols.^34^ Subsequently, two kinds of polymer ligands with different end groups, that is, thiol-terminated poly(ethylene oxide) and carboxyl-terminated poly(ethylene oxide), were grafted to the surface of Au and Al NPs, respectively.^35^ Using the NHS ester cross-linking reaction, the Cy5 fluorescent molecule (Cy5-NHS) was modified onto the surfaces of Au and Al NPs. Specifically, the Cy5-NHS (0.1 mg/ml, 2 ml) and polymers (2.0 mg) were added into pH 8.0 phosphate buffer and stirred at 4 °C for 12 h.

### C. Cell culture and cytotoxicity assay

Cells were grown in Dulbecco's Modified Eagle's Medium (DMEM, Gibco) supplemented with 10% fetal bovine serum (FBS, TransGen Biotech) or house serum (Gibco) and 1% streptomycin and penicillin at 37 °C with 5% CO_2_ humidified atmosphere.

The cytotoxicity of Au and Al NPs was evaluated via an LDH cytotoxicity assay kit (Beyotime Biotechnology) based on the manufacturer's instructions. B16 cells were seeded into 96-well plates (10^4^ cells/well). Subsequently, Al and Au NPs (at different concentrations) were added to the cells. Cells cultured in a complete medium without particles were used as the control. At each time point, 200 *μ*l of the medium was measured at wavelengths corresponding to 490 nm. Background absorbance values (Abs) at 600 nm were subtracted, and data were converted to percent cytotoxicity using the following formula: [Abs of sample − Abs of control]/[Abs of maximum release sample − Abs of control] × 100. Each condition was analyzed in triplicate.

### D. Cellular uptake assay

B16 or NCTC1469 cells were seeded into 24-well plates (10^5^ cells/well) and incubated overnight at 37 °C with a 5% CO_2_ humidified atmosphere. Nanoparticles conjugated with Cy5 in complete DMEM were added into each well. After co-incubation at different times, the cells were analyzed by flow cytometry (LSRFortessa, BD Bioscience, San Jose, CA, USA). The data were analyzed by FlowJo software (TreeStar, San Carlos, CA, USA).

### E. Animals and tumor model

Female C57BL/6 mice (7 weeks old) were purchased from Charles River Laboratories (Beijing, China). All mice were raised in a specific pathogen-free environment with free access to food and water. All animal protocols were reviewed and approved by the Institutional Animal Care and Use Committee of the First Hospital of Jilin University, and all experiments were performed in accordance with the protocols.

B16 cells (1 × 10^6^) were administered by subcutaneous injection into the backs of mice to set up a tumor-bearing mouse model.

### F. Tissue distribution and ICP-MS quantification of nanoparticle retention

When the tumor volume was about 200–300 mm^3^, either Cy5-Al NPs, Cy5-Au NPs, or PBS was intravenously injected into the tumor-bearing mice. The IVIS system (PerkinElmer, Waltham, MA, USA) detected the Cy5 signal in tumors and major organs (brain, lung, heart, liver, spleen, and kidneys). Data were analyzed by Living Image software (PerkinElmer, Waltham, MA, USA).

Major organs (brain, lung, heart, liver, spleen, and kidneys) and tumors for ICP-MS analysis were rapidly harvested at 24 h and 7 days following nanoparticle injection. Then the harvested tissues were washed with PBS and dried, they were cut into several 100–200 mg pieces, and weighed. Nitric acid (0.50 ml) and hydrogen peroxide (20 *μ*l) were added to each sample, and then reacted at 120 °C to nitrolysis. Homogenized samples were thoroughly mixed with aqua regia [nitric acid and hydrochloric acid (1:3, v/v)] for 3 days. De-ionized water (10 ml) was added and filtered twice and subjected to ICP-MS analysis (iCAP Q, Thermo Fisher, Waltham, MA, USA).

### G. Blood sampling and analysis

For serum collection, blood was collected from tail vein of mice. The following biochemical parameters were then evaluated using specific assay kits (Jiancheng Bioengineering Institute, China): (1) alanine aminotransferase (ALT) and aspartate aminotransferase (AST) for liver function; and (2) blood urea nitrogen (BUN) and creatinine (CRE) for kidney function, in a multifunctional microporous plate autoanalyzer (Spark, TECAN, Mannedorf, Zurich, Switzerland).

### H. Histopathology

The main organs (brain, lung, heart, liver, spleen, and kidneys) and tumors were harvested and stained with H&E. The sections were observed through an optical microscope (IX71, Olympus, Tokyo, Japan).

### I. Statistical analysis

All data are presented as mean ± standard deviation. Parametric data were statistically analyzed via a one-way ANOVA test using Graph Pad Prism (version 8.0). A p-value < 0.05 was considered statistically significant.

## SUPPLEMENTARY MATERIAL

See the supplementary material for additional biomaterial characterization data and cytotoxicity data.

## Data Availability

The data that support the findings of this study are available within the article and its supplementary material.
